# Extending Hilbert–Schmidt Independence Criterion for Testing Conditional Independence

**DOI:** 10.3390/e25030425

**Published:** 2023-02-26

**Authors:** Bingyuan Zhang, Joe Suzuki

**Affiliations:** Graduate School of Engineer Science, Osaka University, Toyonaka 560-0043, Japan

**Keywords:** conditional independence test, dependence measure, local bootstrap

## Abstract

The Conditional Independence (CI) test is a fundamental problem in statistics. Many nonparametric CI tests have been developed, but a common challenge exists: the current methods perform poorly with a high-dimensional conditioning set. In this paper, we considered a nonparametric CI test using a kernel-based test statistic, which can be viewed as an extension of the Hilbert–Schmidt Independence Criterion (HSIC). We propose a local bootstrap method to generate samples from the null distribution $H_{0}:X⫫Y|Z$. The experimental results showed that our proposed method led to a significant performance improvement compared with previous methods. In particular, our method performed well against the growth of the dimension of the conditioning set. Meanwhile, our method can be computed efficiently against the growth of the sample size and the dimension of the conditioning set.

## 1. Introduction

The Conditional Independence (CI) test is a statistical hypothesis test that examines whether variables *X* and *Y* are conditionally independent given another variable *Z*, denoted as X⫫Y∣Z, when we observe the actual values of the three variables. The CI test plays a critical role in Bayesian network structure learning [1,2] and causal discovery [3].

The task is relatively easy when the sample size *n* is large and the variable *Z* is discrete, because then, we can test the independence of X,Y for each value of *Z* [4]. On the other hand, if X,Y,Z have a joint Gaussian distribution, then the CI reduces to a zero partial correlation between *X* and *Y* given *Z* [5], which can also be easily tested. In this paper, we considered X,Y,Z without making any assumption on the joint distribution. X,Y,Z can be either continuous or discrete variables. The problem becomes challenging with a growing dimension $d_{Z}$ due to the curse of dimensionality [6], when *Z* may be a set of $d_{Z}$ variables or any $d_{Z}$-dimensional random vector.

Another major challenge in CI tests is the need to sample from the null distribution $H_{0}:X⫫Y|Z$. In general, statistical hypothesis tests require us to obtain the distribution of the test statistic under the null hypothesis $H_{0}$. However, when we are only given the observations, the exact distribution for any test statistic under the CI case ($H_{0}:X⫫Y|Z$) is unknown. The two approaches below are the most-popular ways to obtain an approximated null distribution:

• **Permutation method:**
  One approach is by permuting the observed samples. In the independence test, where $H_{0}:X⫫Y$, though *X* and *Y* in each pair $\left(x_{1},y_{1}\right),\ldots ,\left(x_{n},y_{n}\right)$ are not independent, we may regard *X* and *Y* of shifted pairs of $(x_{1},y_{2}),\ldots ,$$\left(x_{n-1},y_{n}\right),\left(x_{n},y_{1}\right)$ to be independent. Thus, we can compute the test statistic values on the shifted pairs, which mimic $H_{0}$, and obtain a histogram as an approximated null distribution. However, in the CI test, as the conditioning set *Z* exists, we cannot shift $\left\{x_{i}\right\},\left\{y_{i}\right\},\left\{z_{i}\right\}$ in order to make them conditionally independent [7,8].
• **Asymptotic method:**
  The other approach utilizes the asymptotic distributions of the test statistics [9,10,11]. For some test statistics, their asymptotic distributions are derived. In that case, the asymptotic distribution of a test statistic can be used to approximate the null distribution. Though these asymptotic distributions can be generated efficiently, they are less accurate when the sample size *n* is small or with a high-dimensional *Z* [8,12].

*Our contributions*: In this paper, we propose a new CI test including a novel test statistic and a local bootstrap method to sample from $H_{0}:X⫫Y|Z$. In many CI tests, test statistics directly evaluate the conditioning set *Z*, which becomes difficult when *Z* is high-dimensional or has a complex density. Our proposed test statistic does not directly access conditioning set *Z*, which alleviates the curse of dimensionality. Such a test statistic is expected to be more robust for a high-dimensional conditional set. The experiment result showed that our proposed test had a comparable performance when *Z* is low-dimensional and notably outperformed others when *Z* is high-dimensional. Moreover, our proposed method can be computed efficiently regarding the growing sample size *n* and growing dimension of *Z*. We summarize our main contributions as follows:

• We designed a novel test statistic in the following procedure: we first subdivided *Z* into several local clusters, then measured the unconditional independence in each cluster, and finally, combined the unconditional independence measures into a single number as the measure of conditional independence. In particular, we used *k*-means to find clusters of *Z* and the Hilbert–Schmidt Independence Criterion (HSIC) [13] as the measure of unconditional independence in each cluster. We took the sum of the local HSIC values as our test statistic for conditional dependence.
• We propose to use a local bootstrap method to sample from the CI case $H_{0}:X⫫Y|Z$. We extended the local bootstrap strategy in [14] and showed the theoretical consistency of the bootstrap distribution. The local bootstrap method worked well combined with the proposed test statistic, but can also be applied to other CI tests.

The paper is organized as follows. In Section 2, we discuss some related works on the CI test. In Section 3, we introduce the notations and provide an overview of the HSIC, a kernel-based measure of unconditional independence. In Section 4, we show the details about the test procedure and explain both the test statistic and the local bootstrap method. In Section 5, we compare with other representative CI tests based on the synthetic data. Finally, we summarize our results in Section 6.

## 2. Related Work

Recently, numerous nonparametric methods have been proposed for CI testing. Many test statistics have been constructed by embedding distributions in Reproducing Kernel Hilbert Spaces (RKHSs). Fukumizu et al. [7] proposed a measure of CI based on cross-covariance operators. However, its asymptotic distribution under the null hypothesis is unknown, and the bin-based permutation degrades as the dimension of conditioning variable *Z* grows. Later, Zhang et al. [10] proposed the KCIT, based on the partial association of functions in some universal RKHS. A major advantage of the KCIT is a known asymptotic distribution that can be efficiently approximated using Monte Carlo simulations. For the CI test on a large-scale dataset, Strobl et al. [11] proposed the RCIT and RCoT to use random Fourier features to approximate the KCIT efficiently. Huang et al. [15] proposed a Kernel Partial Correlation (KPC), a generalization of a partial correlation to measure conditional dependence. Beyond kernel-based methods, Runge [12] used a Conditional Mutual Information (CMI) estimator as the test statistic and proposed a k-nearest-neighbor-based permutation to generate samples from the null distribution. Shah and Peters [16] proposed a Generalized Covariance Measure (GCM) as the test statistic based on regression method. Doran et al. [8] turned the CI test into a two-sample test by finding a permutation matrix and measuring the Maximum Mean Discrepancy (MMD) [17] between the two distributions. Sen et al. [18] proposed a method called the CCIT, which turns the CI test into a classification problem. In [8,18], they both gave an additional sampling step involving data splitting, potentially reducing the power when the dataset is small. Some other model-powered methods also make use of the GAN [19,20] and Double-GAN [21].

While nonparametric CI tests make no assumption about the joint distribution of X,Y,Z, imposing additional assumptions helps to simplify the problem. Some milder assumptions are considered. In particular, *X* and *Y* are assumed to be in function forms of variable *Z* plus an additive independent noise term, which has a zero mean:

$$X=f\left(Z\right)+\varepsilon_{x},\;Y=g\left(Z\right)+\varepsilon_{y}.$$

If the estimated noise terms are independent $\varepsilon_{x}⫫\varepsilon_{y}$, we conclude that X⫫Y∣Z [22,23,24,25,26]. The methods in this category need to find a regression function and then test for the unconditional independence of the residuals.

For further details about the different characterizations of CI, see [27]. From a theoretical perspective, Shah and Peters [16] proved there exists no universally valid CI testing for all CI cases. Precisely, no CI test can control Type-I error for all the CI cases while having a higher power against any alternative. However, a desirable CI test is supposed to be computationally efficient.

## 3. Background on Kernel Methods

This section introduces the notations and gives the basic definitions related to the kernel methods. For further details, see [13,28,29]. We use X,Y,Z and x∈X,y∈Y,z∈Z to represent random variables and their observed samples and use X,Y,Z to denote the associated domains. We considered a positive definite kernel k:X×X→R that corresponds to a Hilbert space H and a feature map Ψ:X→H such that

$$k\left(x_{1},x_{2}\right)={\langle \Psi \left(x_{1}\right),\Psi \left(x_{2}\right)\rangle }_{H}$$

for $x_{1},x_{2}\in X$, where ${\langle \cdot ,\cdot \rangle }_{H}$ is the inner product of the Hilbert space H. Such an H is a Reproducing Kernel Hilbert Space (RKHS) with respect to the kernel *k*, denoted as $H_{k}$. For example, the Gaussian kernel $k\left(x_{1},x_{2}\right)=\exp\left(-∥x_{1}-x_{2}{∥}^{2}/\sigma \right)$ is a positive definite kernel, and we considered it a default choice in the paper. Let *k* be a kernel defined on X and its corresponding RKHS be $H_{k}$. We fixed a set P of measures.

**Definition** **1** (Kernel embedding). *The kernel embedding of the measure μ into the RKHS $H_{k}$ is the map $m_{k}:P\to H_{k}$ defined by*

$$P∋\mu \mapsto m_{k}(\mu ):=\int k\left(\cdot ,x\right)d\mu \left(x\right)\in H_{k}\;.$$

From the above definition, a direct consequence is

$$\int f\left(x\right)d\mu \left(x\right)={\langle f,m_{k}\left(\mu \right)\rangle }_{H_{k}},\forall f\in H_{k}.$$

**Definition** **2** (MMD). *The Maximum Mean Discrepancy (MMD) between P,Q∈P is*

$$MMD\left(P,Q\right):=∥m_{k}\left(P\right)-m_{k}{\left(Q\right)∥}_{H_{k}}^{2}.$$

It is easy to see that the MMD takes non-negative values. In particular, for characteristic kernels (e.g., the Gaussian kernel), the MMD(P,Q) becomes zero if and only if the measures P,Q coincide [17].

Finally, we considered an unconditional dependence measure for variables *X* and *Y*. Let $k_{X}$ and $k_{Y}$ be kernels on X and Y and $H_{k_{X}}$ and $H_{k_{Y}}$ be the corresponding RKHSs. Gretton et al. [13] defined the Hilbert–Schmidt Independence Criterion (HSIC), which can be viewed as the MMD between a measure $P_{XY}$ of X,Y and the product $P_{X}P_{Y}$ of the marginalized measures $P_{X},P_{Y}$. The HSIC is a state-of-the-art dependence measure, which suits both continuous and discrete variables. The HSIC has been well studied as a test statistic in independence testing [10,13,17]. For a characteristic kernel, the HSIC(X,Y) is zero if and only if $P_{XY}=P_{X}P_{Y}$, which indicates X⫫Y.

More precisely, we may express the HSIC as follows:

**Definition** **3** (HSIC).

$$\begin{array}{cc} HSIC(X,Y):= & \;∥m_{k}-m_{k_{X}}m_{k_{Y}}{∥}_{H}^{2}\\ = & \;∥E_{XY}\left[k_{X}\left(X,\cdot \right)k_{Y}\left(Y,\cdot \right)\right]-E_{X}\left[k_{X}\left(X,\cdot \right)\right]E_{Y}\left[k_{Y}\left(Y,\cdot \right)\right]{∥}_{H}^{2}, \end{array}$$

*where H is the corresponding RKHS of the kernel $k:=k_{X}k_{Y}$ defined by*

$$k\left(\left(x,y\right),\left(x^{\prime },y^{\prime }\right)\right)=k_{X}\left(x,x^{\prime }\right)k_{Y}\left(y,y^{\prime }\right)$$

*for $\left(x,y\right),\left(x^{\prime },y^{\prime }\right)\in X\times Y$.*

The HSIC(X,Y) is known to have an alternative expression:

(1) $$\mathrm{HSIC}\left(X,Y\right)=E_{XYX^{\prime }Y^{\prime }}\left[C\left(X,Y,X^{\prime },Y^{\prime }\right)\right]$$

where $C(X,Y,X^{\prime },Y^{\prime })$ is

(2) $$\begin{array}{cc} \; & \left[k_{X}\left(X,X^{\prime }\right)-E_{X^{\prime \prime }}\left[k_{X}\left(X,X^{\prime \prime }\right)\right]\right]\left[k_{Y}\left(Y,Y^{\prime }\right)-E_{Y^{\prime \prime }}\left[k_{Y}\left(Y^{\prime },Y^{\prime \prime }\right)\right]\right], \end{array}$$

and $\left(X^{\prime },Y^{\prime }\right)$ are independent copies of (X,Y). Given data points $\left(x_{1},y_{1}\right),\ldots ,\left(x_{n},y_{n}\right)$, we considered the following estimator [13]:

(3) $$\hat{\mathrm{HSIC}}\left(X,Y\right)=\frac{1}{n^{2}}\mathrm{tr}\left(K_{X}HK_{Y}H\right)$$

where ${\left(K_{X}\right)}_{ij}=k\left(x_{i},x_{j}\right),{\left(K_{Y}\right)}_{ij}=k\left(y_{i},y_{j}\right)$, $H=I-\frac{1}{n}𝟙{𝟙}^{T}$, and 𝟙 is an *n* vector of ones. Intuitively, we expect an estimator of the HSIC to be a small value when X⫫Y.

## 4. Proposed Method

In this section, we introduce our proposed method. First, we present a novel test statistic. We considered using characteristic kernels as a default choice, i.e., the Gaussian kernel. Next, we explain the local bootstrap algorithm to generate samples from $H_{0}:X⫫Y|Z$. The test is summarized in Algorithm 1. Finally, we discuss the effect of the parameters and provide a time complexity analysis of the overall procedure.

We start by looking at the CI definition: X⫫Y∣Z means *X* and *Y* are independent for any fixed value of *Z*. Here, we used $\mathrm{HSIC}\left(X,Y|Z=z\right):=E_{XYX^{\prime }Y^{\prime }}\left[C\left(X,Y,X^{\prime },Y^{\prime }\right)|Z=z\right]$ to represent the HSIC on (X,Y) with a fixed *Z* value, where $\left(X^{\prime },Y^{\prime }\right)$ are copies of (X,Y).

$$\begin{array}{cc} X⫫Y|Z\Leftrightarrow & X⫫Y|Z=z,\;\forall z\in Z.\\ \Leftrightarrow \mathrm{HSIC}(X,Y|Z=z)=0,\;\forall z\in Z. \end{array}$$

As a direct result, we have the following proposition.

**Proposition** **1** (Characterization of CI).

(4) X⫫Y∣Z⇔∫HSIC(X,Y∣Z)dμ(Z)=0

*where μ(Z) is the probability measure on Z.*

*Proof sketch*: By definition, HSIC(X,Y∣Z=z)=0 always takes non-negative values. Thus, for a characteristic kernel, the integral becomes zero if and only if HSIC(X,Y∣Z=z)=0,∀z∈Z, which indicates X⫫Y∣Z.

Based on the above fact, conditional dependence can be measured by the marginal unconditional dependence measure. Here, we considered the following procedure to calculate our test statistic:

1. Perform the clustering algorithm to subdivide *Z* into *M* clusters, and let its index set be $C_{M}$.
2. Measure the unconditional dependence ${\hat{\mathrm{HSIC}}}_{C_{m}}\left(X,Y\right)$ for each cluster $C_{m}$.
3. Combine the sum of the values as the test statistic:
  (5) $$T=\sum_{m=1}^{M}{\hat{\mathrm{HSIC}}}_{C_{m}}\left(X,Y\right).$$

We used the sum of the local unconditional dependence measure as the conditional dependence measure, which is similar in spirit to [4]. Margaritis [4] considered dividing a univariate $Z\in R^{1}$ into local bins and using the product of the local measure as a single number. Our method applies to a high-dimensional *Z* and takes the sum of kernel-based measures. Given the data $(x_{i},y_{i},z_{i}),i=1,\ldots ,n$, we divided them into *M* clusters based on the value of *Z*, and the estimator is

$${\hat{\mathrm{HSIC}}}_{C_{m}}\left(X,Y\right)=\frac{1}{|C_{m}{|}^{2}}\mathrm{tr}\left(K_{X}^{\left(m\right)}HK_{Y}^{\left(m\right)}H\right)$$

where $|C_{m}|$ is the size of $C_{m}$ and $K_{X}^{\left(m\right)}$ and $K_{Y}^{\left(m\right)}$ are the corresponding kernel matrices for samples $\left(x_{i},y_{i}\right),\;\forall i\in C_{m}$. It is easy to see that the conditioning set *Z* is only used in deciding the local clusters. By doing that, we alleviate the influence of the dimension of *Z*.

### 4.1. Local Bootstrap

**Figure 1:**
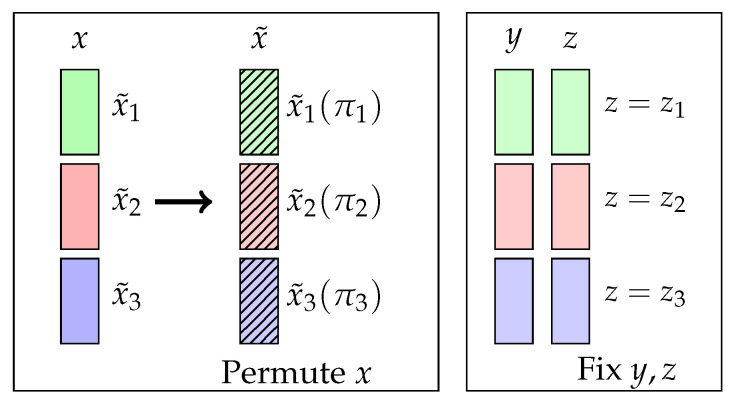
An ideal permutation in the CI. Given the data, we first divided different bins (green, red, and blue), and each bin $\tilde{x}$ includes samples that have the same *z*. From that, we fixed *y* and *z* and shuffled *x* within each bin with some permutations ($\pi_{1},\pi_{2},\pi_{3}$) to generate new data $\left(\tilde{x},y,z\right)$. An ideal permutation successfully generates samples that keep the dependence between (X,Z) and (Y,Z) while satisfying X⫫Y.

In this subsection, we introduce the local bootstrap method to sample from $H_{0}:X⫫Y|Z$, which completes the CI test. The key is to break the dependence between *X* and *Y* while keeping the dependence between (X,Z) and (Y,Z). An example of an ideal CI permutation is explained in Figure 1.

In practice, it is impossible to perform the ideal permutation because we do not have enough samples that have the same *z*. As an alternative method, we used a local bootstrap.

First, given with different $z^{*}$, we generated $\left(x^{*},y^{*}\right)$ independently from the following discrete distribution:

(6) $$\begin{array}{cc} \; & x^{*}∼{\hat{G}}_{x|z^{*}}:\left(\begin{array}{cccc} x_{1} & x_{2} & \ldots , & x_{n}\\ w_{1} & w_{2} & \ldots , & w_{n} \end{array}\right),\\ \; & y^{*}∼{\hat{G}}_{y|z^{*}}:\left(\begin{array}{cccc} y_{1} & y_{2} & \ldots , & y_{n}\\ w_{1} & w_{2} & \ldots , & w_{n} \end{array}\right), \end{array}$$

where $w_{j}=\frac{K(z_{j}-z^{*}/\gamma )}{\sum_{j=1}^{n}K\left(z_{j}-z^{*}/\gamma \right)}$ are the probabilities to sample the index *j*. Under the mild assumptions [30], we show the consistency of the bootstrap distribution at each $z^{*}$. See the proof in Appendix A.

**Proposition** **2.** The empirical bootstrap distribution converges to a conditional distribution with each fixed point of $z^{*}$:

(7) $$|{\hat{G}}_{x|z^{*}}{\hat{G}}_{y|z^{*}}-P\left(X|Z=z^{*}\right)P\left(Y|Z=z^{*}\right)|\to 0,\;as\;n\to \infty .$$

The local bootstrap strategy is an extension of [14], which was original designed for sampling (x,y) according to a regression model. We extended it using a Nadaraya–Watson kernel estimator [31] to assign the weights for indexes to be sampled. If $z_{j}$ is close to $z^{*}$, $w_{j}$ is large; thus, the index *j* has a larger possibility of being sampled. Moreover, $x^{*}$ and $y^{*}$ are sampled independently. Thus, it is less possible for both $x_{j}$ and $y_{j}$ to be sampled simultaneously, which breaks the dependence between *X* and *Y*. Shi [14] suggested that the bandwidth γ should be varied for different $z^{*}$. Here, we narrowed the candidates from 1,…,n to the 10-nearest neighbors of each $z^{*}$ and let the local bandwidth γ of $z^{*}$ be the squared Euclidean distance between $z^{*}$ and its 10-th nearest neighbor.

The local bootstrap is summarized in Algorithm 1. Each time, we generated *n* samples as if they come from $H_{0}:X⫫Y|Z$ and calculated the test statistic value *T*. We repeated this *K*-times on the generated samples and calculated the *p*-value based on the histogram. We reject $H_{0}$ if the *p* value is smaller than a predefined significance level. Otherwise, we accept $H_{0}$. We summarize the procedure in Algorithm 2.

**Algorithm** **1:**Local bootstrap
**Input**: Data $\left(x_{i},y_{i},z_{i}\right)$, i=1,…,n.
**Output**: New samples: $(x_{i}^{*},y_{i}^{*},z_{i}^{*}),i=1,\ldots ,n$
1
**for** i←1 **to** *n* **do**
2     Let $z_{i}^{*}=z_{i}$3     Sample $x_{i}^{*}∼{\hat{G}}_{x|z_{i}^{*}},\;y_{i}^{*}∼{\hat{G}}_{y|z_{i}^{*}}$.4
**end**



**Algorithm** **2:**Test
**Input**: Data $(x_{i},y_{i},z_{i}),i=1,\ldots ,n$.
            Cluster number *M*.
            Times to repeat *K*.
**Output**: *p*-value.
1Find *M* clusters.2Estimate the *T*.3
**for** k←1 **to** *K* **do**
4     Generate samples with Algorithm 15     Estimate $T_{i}$ on the generated samples.6
**end**
7Compute the *p*-value:
$$p=\frac{1}{K}\sum_{t=1}^{T}\{T_{k}\geq T\}.$$


### 4.2. Effect of *M*

The choice of the cluster number *M* affects the test performance. *M* needs to be chosen properly so that we can focus on the pairs (x,y) who have similar *z* values while having enough pairs (x,y) in each local cluster to make a good estimation of the local HSIC. Besides, the number of *M* has an effect on the computational complexity: a smaller *M* makes bigger clusters on average and takes more time to find local HSICs. In practice, we fixed the average cluster size and used *k*-means to decide the clusters. We let *M* be $\left\lceil n/\bar{C}\right\rceil$, where ⌈x⌉ takes the least integer that is not smaller than *x*, and $\bar{C}$ is the average cluster size.

### 4.3. Complexity Analysis

We discuss the time complexity of the test procedure. In the beginning, our method found *M* clusters and weights *w* in the bootstrap. Both were calculated once and took little time. The major computational cost was in repeatedly finding the test statistic $T_{k}$. Estimating *T* scales less than $O(M|\tilde{C}{|}^{2})$, where *M* is the number of clusters and the $|\tilde{C}|$ is the maximum set size among all clusters and is smaller than *n*. This was repeated *T*-times over the generated samples to construct the histogram of the null distribution. The test took $O(Mn^{2}K)$. The bootstrap part can be easily parallelized to promote the speed further, but this was beyond the scope of the paper.

## 5. Experiments

In this section, we compare the proposed methods with other nonparametric CI tests. We denote our proposed method as a Bundle of HSICs (**BHSIC**). The evaluation was focused on the Type-I error rate, Type-II error rate, and runtime. A lower Type-II error rate and computational efficiency are essential for a good CI test. In particular, we compared with some representative methods: **KCIT** [10], **RCIT, RCoT** [11], **CCIT** [18], and **CMIknn** [12]. For details about these methods, see Section 2. All methods have source codes that are available online. Different methods are implemented in different programming languages, and we focused on how these methods scale with the sample size and the dimension of *Z* instead of a direct comparison of the runtimes.

We were interested in the performance of the methods with different settings. In our simulations, we considered the following two models. The first model was a simple linear regression model. The second model was a post-nonlinear noise model, which is a commonly used setting in evaluating CI tests [10,11,12]. The functional forms of *X* and *Y* on *Z* are as follows:

$$\begin{array}{cc} \mathrm{Model}\;1: & \;X=\sum_{i=1}^{d_{Z}}\alpha_{i}Z_{i}+c\varepsilon_{b}+\varepsilon_{1},\;Y=\sum_{i=1}^{d_{Z}}\beta_{i}Z_{i}+c\varepsilon_{b}+\varepsilon_{2},\\ \mathrm{Model}\;2: & \;X=g_{1}\left(\sum_{i=1}^{d_{Z}}Z_{i}+c\varepsilon_{b}+\varepsilon_{1}\right),\;Y=g_{2}\left(\sum_{i=1}^{d_{Z}}Z_{i}+c\varepsilon_{b}+\varepsilon_{2}\right), \end{array}$$

where $Z=(Z_{1},\ldots ,Z_{d_{Z}})$, $\varepsilon_{1}$, $\varepsilon_{2}$, and $\varepsilon_{b}$ are independent standard Gaussian. The coefficients $\alpha_{i},\beta_{i}∼Uniform\left(-0.5/d_{z},0.5/d_{z}\right)$ and the functions $g_{1}\left(\cdot \right)$ and $g_{2}\left(\cdot \right)$ were uniformly chosen from $\{\left(\cdot \right),{\left(\cdot \right)}^{2},{\left(\cdot \right)}^{3}$, tanh(·), $\exp(-∥\cdot {∥}_{2})\}$. We considered (a) $H_{0}:X⫫Y|Z$ with c=0 and (b) $H_{1}:$X⫫Y∣Z with c=1.

In the following simulations, we studied the test performance on different sample sizes and dimensions of *Z*. The sample sizes *n* varied from {100,200,400,600,800} with fixed dimensions of $d_{Z}=1$ and $d_{Z}=10$. The dimensions $d_{Z}$ varied from {1,2,5,10,20} with a fixed sample size of n=400. We also studied the effect of the cluster number *M* in our proposed method. The significance levels were set to be α=0.05 in all the simulations. The evaluations of the Type-I error rate, Type-II error rate, and mean runtimes are reported over 100 replications. The Type-I error rate is the false rejection percentage when the underlying truth is $H_{0}:X⫫Y|Z$ with c=0, and the Type-II error rate is the false acceptance percentage when the underlying truth is $H_{1}:$X⫫Y∣Z with c=1. Runtime is the average time to perform one test.

### 5.1. Hyperparameters

The choice of the hyperparameters affects the results. For the KCIT, RCIT, and RCoT, the bandwidths in the Gaussian kernels were set to be the squared median Euclidean distance between (X,Y) using all the pairs (or the first 500 pairs if n>500) double the conditioning set size, which was recommended in [11]. Thew CMIknn has two hyperparameters: the neighbor size $k_{\mathrm{CMI}}=0.1n$ in finding the estimator of the CMI, and $k_{perm}=5$ in the permutation, respectively. The permutation in CMIknn was repeated 1000-times as the default [12].

In our proposed methods, the bandwidths were set to be the squared median Euclidean distance between (X,Y) in each local cluster. The number of clusters *M* was set to be ⌈n/50⌉ when n<=200 and ⌈n/80⌉ when n>200, where ⌈x⌉ takes the least integer that is bigger than or equal to *x*. On average, each cluster had 50 samples when n<=200 and 80 samples otherwise. The local bootstrap was repeated 1000-times.

### 5.2. When Z Is Low-Dimensional

**Figure 2:**
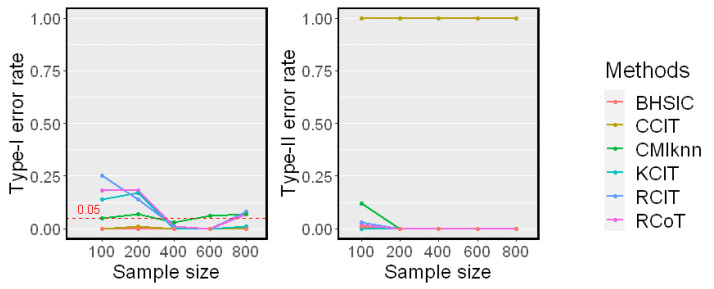
Simulation results on Linear Model 1 ($d_{Z}=1$). The significance level is α=0.05. Type-I error rates and Type-II error rates are reported.

**Figure 3:**
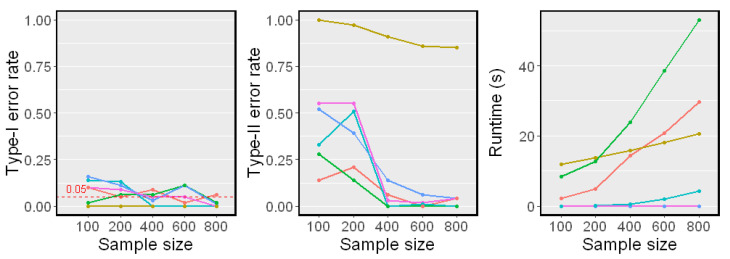
Simulation results on Nonlinear Model 2 ($d_{Z}=1$). The significance level is α=0.05. Type-I error rates, Type-II error rates, and mean runtimes are reported.

We first examined the test performance when *Z* is generated independently of a standard Gaussian distribution. The sample size *n* changed from 100 to 800. The simulation results on Linear Model 1 and Nonlinear Model 2 are reported in Figure 2 and Figure 3, respectively. Both the Type-I error rate and Type-II error rate are reported. Because runtime is independent of the model, we only report it in Figure 3.

The Linear Model 1 setting with a single conditioning variable *Z* is very simple. All methods had controlled Type-I error rates around α=0.05 and almost zero Type-II error rates, except for the CCIT. In our experiments, the performance of the CCIT was constantly among the worst. The data splitting procedure in the CCIT seems to reduce the power of the test when the sample size is small. In the Nonlinear Model 2 setting, all methods had controlled Type-I error rates around α=0.05. However, it was shown that the proposed method and the CMIknn had better powers against the others when the sample size *n* was smaller. This matched the result in [12] that CMIknn performed well with a low-dimensional conditioning set *Z*. When the sample size *n* was larger than 400, most methods had relatively low Type-II error rates. From the runtime plot, the proposed method was less efficient than the KCIT, RCIT, and RCoT, which are based on an asymptotic distribution. Though the BHSIC and CMIknn were slower, the sampling procedure can readily be parallelized.

### 5.3. When Z Is High-Dimensional

**Figure 4:**
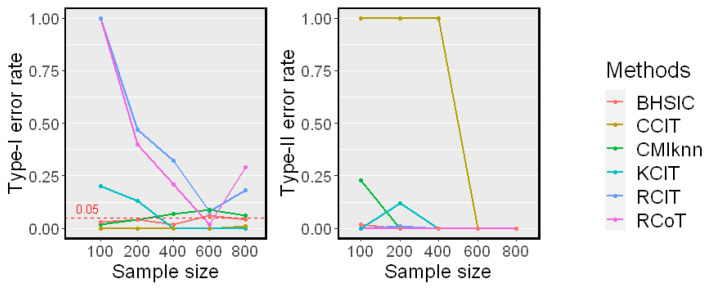
Simulation results on Linear Model 1 $\left(d_{Z}=10\right)$. The significance level α=0.05. Type-I error rates and Type-II error rates are reported.

**Figure 5:**
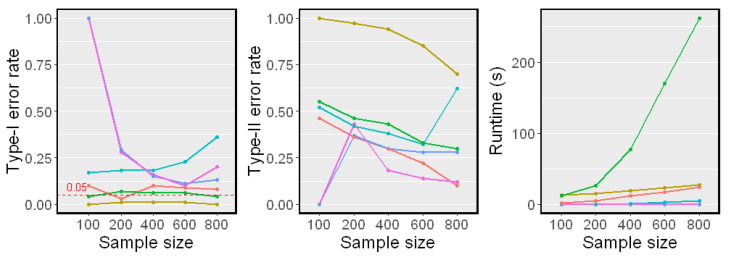
Simulation results on Nonlinear Model 2 $\left(d_{Z}=10\right)$. The significance level α=0.05. Type-I error rates, Type-II error rates, and mean runtimes are reported.

We next examined the test performance when *Z* was a set of 10 variables, and each variable in conditioning set *Z* was generated independently from a standard Gaussian distribution. The sample size *n* changed from 100 to 800. The simulation results on Linear Model 1 and Nonlinear Model 2 are reported in Figure 4 and Figure 5, respectively.

In both linear and nonlinear settings, the RCIT and RCoT failed and had high Type-I error rates. The RCIT and RCoT approximated the KCIT by using random Fourier features and were designed for large-scale datasets. Though they are more scalable than the KCIT, their performances were poor when the sample size was relatively small. The KCIT, CMIknn, and BHSIC performed well in the linear model setting. In the nonlinear model setting, the KCIT showed greater Type I error rates and Type II error rates because the high-dimensional *Z* led to a less accurate estimation of the asymptotic distribution. We noticed that the BHSIC showed a higher power than the other methods. As we expected, it was beneficial to avoid evaluating the high-dimensional *Z* directly, which made the method more robust.

### 5.4. When $d_{Z}$ Changes

**Figure 6:**
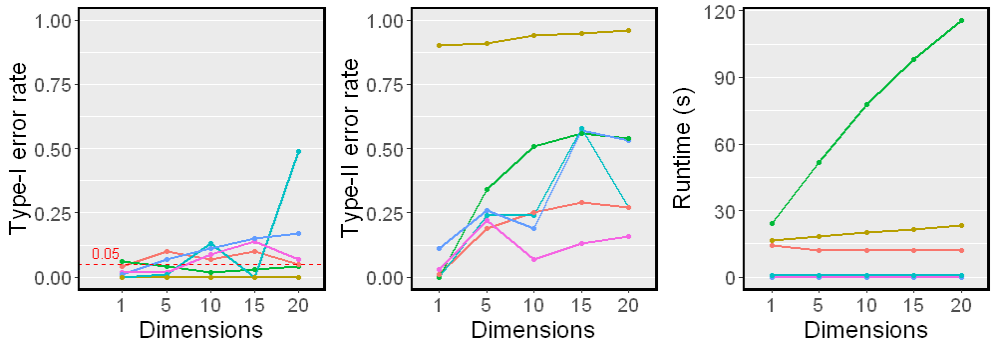
Simulation results on different dimensions of *Z* ($d_{Z}=1,5,10,15,20$). The sample size n=400 was fixed. The significant level α=0.05. Type-I error rates, Type-II error rates, and mean runtimes are reported.

Next, we examined the performance when the dimension of *Z* changes. We fixed the sample size n=400 and changed $d_{Z}$ from 1 to 20 in Nonlinear Model 2. The results are shown in Figure 6. Our proposed method performed well against the growth of the dimension of *Z* and showed a higher power than other methods. Moreover, the dimension of *Z* did not affect the runtimes since *Z* was used in k-means only once, which coincided with our complexity analysis.

### 5.5. Effect on M

**Figure 7:**
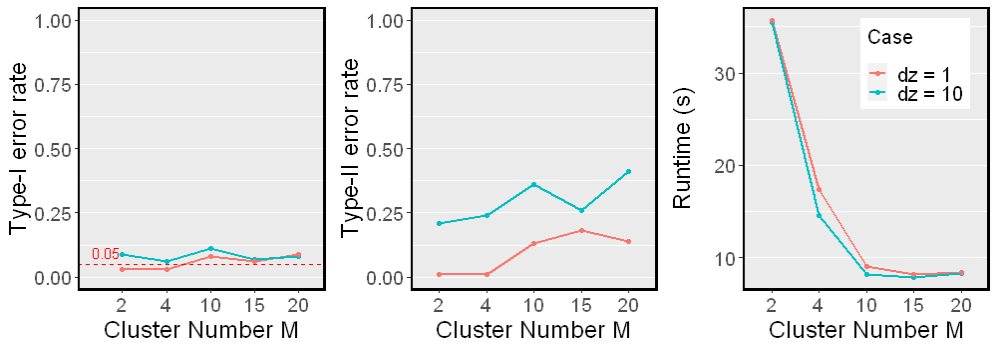
Simulation results on a different cluster number *M*. The sample size n=400 was fixed. Results on different dimensionality of *Z* are reported ($d_{Z}=1$, red line; $d_{Z}=10$, blue line). The significant level α=0.05. Type-I error rates, Type-II error rates, and mean runtimes are reported.

Now, we study the effect on the cluster number *M*. We fixed the sample size n=400 in Nonlinear Model 2 and changed *M* from 2 to 20. We examined both low-dimensional ($d_{Z}=1$) and high-dimensional ($d_{Z}=10$) cases. The results are shown in Figure 7.

We noticed that the Type I error rates were controlled when $d_{Z}=1$ and $d_{Z}=10$. As the cluster number *M* grew to more than 10, the Type-II error rate increased, but the runtimes reduced. The reason is that when we divided the sample points into more clusters, each cluster had fewer points. Thus, the estimation of each local HSIC value became less accurate. On the other hand, the computational cost of the proposed test reduced as the clusters became smaller when the cluster number *M* grew. The number of samples in each cluster depends on the choice of the clustering algorithms as well. In our experiment, we simply used naive k-means.

## 6. Conclusions

In this paper, we proposed a novel CI test including a new test statistic and a local bootstrap method to generate samples from the null hypothesis. We first performed clustering to avoid directly evaluating the high-dimensional conditioning set *Z*. Then, we used the clustering result and combined several local dependence measures as a measure of conditional dependence. Consequently, the problems caused by a high-dimensional *Z* can be suppressed. The experimental results showed that our method is robust and performs well against the growth of the dimension of the conditioning set.

## Data Availability

The evaluation in the paper is based on synthetic data described above.

## Appendix A. Proof of Proposition 2

We assumed that

(A1) $$X=g_{x}\left(Z\right)+\epsilon ,\;Y=g_{y}\left(Z\right)+\epsilon ,$$

and the local bootstrap method generates samples from X⫫Y∣Z.

For each $z_{i}^{*}=z_{i}$, the empirical conditional probability density functions are

(A2) $$\begin{array}{c} {\hat{G}}_{x|z^{*}}\left(x<t\right)=\sum_{j=1}^{n}w_{j}1_{x_{j}<t}, \end{array}$$

(A3) $$\begin{array}{c} {\hat{G}}_{y|z^{*}}\left(y<t\right)=\sum_{j=1}^{n}w_{j}1_{y_{j}<t}. \end{array}$$

where $w_{j}=\frac{K\left(\frac{z_{j}-z^{*}}{\gamma }\right)}{\sum_{j=1}^{n}K\left(\frac{z_{j}-z^{*}}{\gamma }\right)}$. We used $G_{x|z^{*}}$ and $G_{y|z^{*}}$ to denote the underlying truth:

$$\begin{array}{c} G_{x|z^{*}}\left(x<t\right)=P\left(X<t|Z=z^{*}\right),\\ G_{y|z^{*}}\left(y<t\right)=P\left(Y<t|Z=z^{*}\right). \end{array}$$

Consider pairs $\left(x,z\right)\in R\times R^{d}$. Devroye and Wagner (1980) proved the $L^{p}$ convergence of ${\hat{g}}_{x}\left(z\right):=\sum_{j=1}^{n}w_{j}x_{j}$ to $g_{x}\left(z\right)$:

A1 $E[{\left|Z\right|}^{p}]\leq \infty$, p≥1.
A2 γ→0 as n→∞.
A3 $n\gamma^{d}\to 0$ as n→∞.
A4 The kernel *K* satisfies the following:
  − *K* is a nonnegative function on $R^{d}$ bounded by $k^{*}<\infty$.
  − *K* has a compact support *A*.
  − $k\geq \beta 1_{B}$ for some β>0 and some closed sphere *B* centered at the origin with positive radius.

**Lemma** **A1** (Devroye and Wagner, 1980). *Under the above assumption, we have*

(A4) $$E_{X}\left[\int \right|{\hat{g}}_{x}\left(z\right)-g_{x}\left(z\right){|}^{p}d_{z}]\to 0,\;\;\mathrm{as}\;n\to \infty .$$

Let $x_{i}=1_{x_{i}<t}$, and notice that

$$1_{x_{i}<t}=P\left(X_{i}<t\right)+e_{i}$$

where $E(e_{i})=0$ and $\sup_{i}E\left(e_{i}^{k}\right)\leq 1$ for all k≥2. This can be viewed as another non-parametric regression model as in (1). We have

$$E_{X}\left[\int \right|{\hat{G}}_{x|z}-G_{x|z}{|}^{p}dz]\to 0,\;\mathrm{as}\;n\to \infty .$$

Using Lemma 1, we proved the $L^{p}$ convergence of ${\hat{g}}_{x}\left(z\right){\hat{g}}_{y}\left(z\right)$:

**Lemma** **A2.**

(A5)
$$E_{XY}\left[\int \right|{\hat{g}}_{x}\left(z\right){\hat{g}}_{y}\left(z\right)-g_{x}\left(z\right)g_{y}\left(z\right){|}^{p}d_{z}]\to 0,\;\;\mathrm{as}\;\;n\to \infty .$$

**Proof**: 

(A6) $$\begin{array}{cc} \; & \;E_{XY}\left[\int \right|{\hat{g}}_{x}\left(z\right){\hat{g}}_{y}\left(z\right)-g_{x}\left(z\right)g_{y}\left(z\right){|}^{p}d_{z}]\\ = & \;E_{XY}\left[\int \right|{\hat{g}}_{x}\left(z\right)\left\{{\hat{g}}_{y}\left(z\right)-g_{y}\left(z\right)\right\}+g_{y}\left(z\right)\left\{{\hat{g}}_{x}\left(z\right)-g_{x}\left(z\right)\right\}{|}^{p}d_{z}]\\ \leq & \;E_{XY}\left[\int \right|{\hat{g}}_{x}{\left(z\right)|}^{p}|{\hat{g}}_{y}\left(z\right)-g_{y}\left(z\right){|}^{p}d_{z}]\\ \; & +E_{XY}\left[\int \right|g_{y}{\left(z\right)|}^{p}|{\hat{g}}_{x}\left(z\right)-g_{x}\left(z\right){|}^{p}d_{z}]\\ \leq & {\left(k^{*}\right)}^{p}\{E_{Y}\left[\int \right|{\hat{g}}_{y}\left(z\right)-g_{y}{\left(z\right)|}^{p}d_{z}]+E_{X}\left[\int \right|{\hat{g}}_{x}\left(z\right)-g_{x}\left(z\right){|}^{p}d_{z}]\} \end{array}$$

and the right side becomes 0 as n→∞. Similarly, we have

(A7) $$E_{XY}\left[\int \right|{\hat{G}}_{x|z}{\hat{G}}_{y|z}-G_{x|z}G_{y|z}{|}^{p}d_{z}]\to 0,\;\;\mathrm{as}\;\;n\to \infty ,$$

and

$$|{\hat{G}}_{x|z^{*}}{\hat{G}}_{y|z^{*}}-G_{x|z^{*}}G_{y|z^{*}}|\to 0,\;as\;n\to \infty .$$
